# Abuse and disrespect in childbirth process and abortion situation in Latin America and the Caribbean—systematic review protocol

**DOI:** 10.1186/s13643-017-0516-5

**Published:** 2017-08-03

**Authors:** Sofia Madeira, Vicky Pileggi, João Paulo Souza

**Affiliations:** 1 GLIDE Technical Cooperation and Research, Department of Social Medicine - Ribeirão Preto Medical School, University of São Paulo (FMRP/USP), Avenida dos Bandeirantes, 3900, Ribeirão Preto, SP 14049-900 Brazil; 2 Department of Pediatrics of Ribeirão Preto Medical School, University of São Paulo (FMRP/USP), Ribeirão Preto, SP Brazil

## Abstract

**Background:**

Studies show that a large number of women around the world have experienced situations of abuse, disrespect, abuse, and neglect during childbirth and/or abortion. This violence is a serious violation of the rights of women, especially because it is a period in which the woman is more physiologically, socially, and psychologically vulnerable. Although this type of violence is known, there is still no international consensus on the definition of such violence and its prevalence is not known. In this sense, this systematic review aims (1) to find quantitative data about abuse and disrespect in obstetric care (delivery and/or abortion) in Latin America and the Caribbean to estimate the average prevalence of this type of abuse and (2) to identify interventions—including programs, laws, and regulations—which have been implemented to prevent or respond to abuse and disrespect in childbirth process and abortion situation, evaluating its effectiveness on a global scale.

**Methods:**

For this, we will use a refined and pre-established strategy to search databases such as PubMed, Embase, LILACS, and Scielo, and the studies found will pass through a selection process to complete the screening stage.

**Discussion:**

Data will be extracted using standardized forms with the following information: scope of study, sample characteristics, objectives, design, data collection, methods of analysis, data source, and results. Considering the heterogeneity of the definitions of abuse, disrespect, and mistreatment of women in labor or abortion, it may not be possible to carry out the meta-analysis of the frequency of events reported in the included articles. Events reported by the original articles will be classified according to a typology of abuse, disrespect, and maltreatment in the labor or abortion process described by Bohren et al. (PLoS Med, 2015).

**Systematic review registration:**

PROSPERO CRD42016038651

**Electronic supplementary material:**

The online version of this article (doi:10.1186/s13643-017-0516-5) contains supplementary material, which is available to authorized users.

## Introduction

Several international legal instruments have promoted the right to health, including the WHO Constitution and the Universal Declaration of Human Rights. The existence of this law obliges States to create conditions in which citizens access health services. However, ensuring the quality of care of health services has been a major challenge and emerging data suggest that the incidence of abuse and disrespect in childbirth process and abortion situation (Perinatal and Reproductive Health) can be significant. This type of behavior in the perinatal area has been termed as “obstetric violence” and recently as “abuse and lack of respect in gynecology and obstetric care”. The literature which points to the close relationship between such violence and gender contributing to this violation of rights is often ignored. In fact, recent studies show that a large number of women around the world have experienced situations of abuse, disrespect, abuse, and neglect during childbirth and/or abortion. This violence is a serious violation of the rights of women, especially because it is a period in which they are more physiologically, socially, and psychologically vulnerable. Although it is known, there is still no international consensus on the definition of such violence and its prevalence is not known. In this perspective, this systematic review is a relevant effort and initiative of the Pan American Health Organization (PAHO), a regional office of World Health Organization (WHO), to estimate the prevalence of this type of abuse and identify interventions which have been implemented to prevent or respond to abuse and disrespect in childbirth process and abortion situation, evaluating its effectiveness on a global scale.

## Background

### Description and rationale for review question

According to the United Nations Organization (UN, 1948), women are guaranteed the right to full and unrestricted access to sexual and reproductive health and to decent and respectful attention. It means providing a secure and quality service, able to promote access to contraceptive methods and attention to women’s health, especially during the gravid-puerperal cycle. Medical support and institutional care of pregnancy and childbirth eventually reduce maternal mortality, an important indicator of women’s health.

Nevertheless, in recent years, studies show that a large number of women around the world have experienced situations of abuse, disrespect, and neglect during childbirth and/or abortion [1–8]. This violence can be expressed in the form of verbal abuse, humiliation, physical violence, sexual violence, discrimination, neglect, and failure to meet standards of care and attention—such as privacy and confidentiality, limiting access to information, medical procedures without consent, among other ways.

In this perspective, abuse and disrespect represent a violation of the rights of women [5, 9–11], especially because it is a period in which she is more vulnerable physiologically, socially, and psychologically [11]. In addition, mistreatment of women ends up tarnishing the trust relationship established between users and health professionals, serving as a disincentive for future attempts by obstetric care services, affecting both the health of mother and the child [11, 12]. In the face of this situation, WHO has prepared in September 2014 an official statement for prevention and eradication of abuse and disrespect in health institutions worldwide.^1^ As well as other types of violence, abuse and disrespect are not received by women equally, focusing more among women doubly vulnerable by age, social class, ethnicity, for example, adolescents, single women, living in poverty, ethnic minorities, migrants, and HIV positive [13].

Although it is known, there is still no international consensus about the definition of this type of violence [14, 15], its prevalence, impacts, and the whole range of interventions to prevent or even eliminate this problem. In this way, this systematic review of literature is highly suitable for this study, once the systematic methods enable a broader and objective analysis of the produced studies on the subject of interest, providing a conclusive synthesis of the results, and certain interventions also are used to avoid bias [16].

### Systematic review questions

The development of this systematic review will be guided by the following questions:

1. What is the prevalence of abuse and disrespect in childbirth process and abortion situation in Latin America, considering different definitions of this type of violence?

2. What interventions (actions/impacts/results) have been implemented in childbirth process and abortion situation to prevent or respond to such violence on a global scale?

## Aims and objectives

The overall objective of this systematic review is to gather and analyze the literature on the subject of abuse and disrespect in gynecology and obstetric care, in an effort to assess the prevalence of this type of violence and identify interventions implemented to prevent or respond to such violence around the world.

The specific objectives are:

1. Conduct a systematic review to assess the prevalence of abuse and disrespect in childbirth process and abortion situation in Latin America and the Caribbean, encompassing perinatal and reproductive health;

2. Identify interventions—including programs, laws, and regulations—which have been implemented to prevent or respond to abuse and disrespect in childbirth process and abortion situation, evaluating its effectiveness on a global scale.

## Methods

The type and amount of literature available to answer the questions of this systematic review are unknown, requiring that the methods used are modified during the course of this project. In cases where the review team agrees on a particular change in approach, this protocol will be updated to exhibit the alterations, including the reasons for each change made.

### Inclusion and exclusion criteria

Phenomenon of interest:

This systematic review will examine the abusive and/or disrespectful treatment from health professionals received by women in the process of delivery and/or abortion in health institutions.

To better understand the phenomenon of interest, the following table contains the types of abuse and disrespect that will be included in this systematic review [1]—see Additional file 1: Table S1.

Setting:

This review will include studies considering at least one form of abuse or disrespect—whether verbal, physical, and sexual, among others—suffered by women during delivery and/or abortion in health institutions (public or private health institutions that offer hospital medical care to women in childbirth process or abortion); considered as perpetrators of abuse, disrespect, and poor treatment are doctors, nurses, midwives, or other social actors wrapped in traditional or professional assistance to pregnant women and women in labor or abortion situation.

Perspective:

All actors involved in the health system will be included in this review: the users of the services, health professionals, the administrative sector, or managers of public health policies, committed to projects, regiments, and laws capable of promoting interventions for this process of abuse and disrespect in childbirth process and abortion situation. As suggested, different types and levels of health care will be considered.

Comparison group:

Not applicable

The events considered in this systematic review are:

- Prevalence of abuse and disrespect in obstetric and gynecology attendance offered to pregnant women and women in labor or abortion situation in Latin America and the Caribbean
- Possible associations between such violence and health consequences of women and/or child
- Strategies for action and intervention in this scenario of abuse and disrespect and their impact and results
- Raising awareness among health professionals and possible changes in knowledge, attitude, and practice of these professionals
- Reduced frequency of abuse, if found

Studies with the following features will be included:

- Previous systematic reviews and studies of primary or secondary research including characterization or prevalence of abuse and disrespect in gynecology and obstetric care as well as strategies and plans implemented to measure or face such violence laws
- Studies produced recently, from 1990 to the present
- Studies whose full text can be accessed (more than the abstract and keywords)
- For the specific objective 1, only studies in Latin America and the Caribbean will be included
- For the specific objective 2, comprehensive studies that report interventions to reduce abuse and disrespect in childbirth process will be included

Studies with the following characteristics will be excluded:

- Studies that address situations of violence against women which occurred outside the context of childbirth or abortion
- Studies with no explicit methodological contribution, analysis, and data collection
- Studies produced before 1990
- Studies that are not fully accessible
- For specific objective 1, studies conducted outside Latin America and the Caribbean will be excluded
- For specific objective 2, studies that did not try to implement interventions to address abuse and/or disrespect will be excluded

### Language

The language will not be an exclusion criterion in this systematic review. Studies available in Portuguese, Spanish, English, Italian, or French will be evaluated by internal members of the research group; if case studies in different languages are found, we will seek assistance from external partners.

### Information sources

The search strategy of this systematic review is to locate any study potentially relevant—that is, that can assist in the process of understanding and/or response of the guiding questions of this review—from the areas of systems health, medicine, social sciences, and public health.

The following databases will serve as sources of information:

- CINAHL Plus
- Embase
- MEDLINE
- PubMed
- LILACS
- Scielo
- Google*

*Only for objective 2, which will include laws, regulations, and government intervention programs to reduce abuse and disrespect in childbirth process and abortion situation.

The search will be in electronic and manual form, including documents issued or not (gray literature). It includes studies, reports, documents, guides, articles, manuals, laws, and policies, being necessary to investigate reference lists and bibliographies of studies as well as newspapers and magazines around the area of interest—for example, *Reproductive Health Matters*, *The Lancet*, *International Perspectives on Sexual and Reproductive Health*, among others. The review may also include documents produced by PAHO and WHO that have not yet been published, that is, work materials. For this, the review team will make contact with professionals, institutions, research groups, and collaborating centers throughout Latin America to obtain information about publications or unpublished studies on the phenomenon of interest in this systematic review.

### Search strategy

We will develop purpose-specific search strategies for each objective. An outline of the search strategies for objective 1 is found in Annex 1. For objective 2, the strategies will be adapted to include a list of interventions produced by the group INSP/Mexico.

### Stopping rules

Flexible search engines resulting in a very large number of references—such as Google—are subject to stopping rules. In these cases, the search process will cease as follows: when no new literature or large schools of thought related to the issues of the review appears of searches; that is, when the output of potentially eligible items seem exhausted, we will continue making an estimate of the results available just in the five subsequent pages with ten results each, approximately.

### Data handling

Storage and management of bibliographical references will be made by the program Mendeley (https://www.mendeley.com/). Quantitative data will be analyzed by the program REVMAN [17].

### Study selection and data extraction process

Two team members will review independently the titles and abstracts of studies found through searches, using the inclusion and exclusion criteria. The extracted data will be recorded on specific forms and Excel worksheets.

The independent judgements of two reviewers (SM and VP), regarding inclusion and exclusion of studies, will be confronted, and in case of disagreements, a third team member (JPS) will achieve the consensus.

The team will follow a standardized way to extract the main data from the studies found: study design, methodology, characteristics of participants, and issues relevant to the systematic review results.

### Variables of interest

Here are the most important variables of interest in this systematic review, applied in the context of attendance to pregnant women or women in labor in the process of birth or abortion:

- Physical abuse
- Sexual abuse
- Verbal abuse
- Stigma and discrimination
- Failure in compliance with standards and professional standards of care and attention
- Problems between patients and health care providers (communication/trust)

### Evaluation of the quality of the included studies

- The primary studies will be evaluated according to the Critical Appraisal Skills Programme (CASP) and a set of criteria adapted from Cochrane Effective Practice and Organization of Care Review Group (EPOC) Data Collection Checklist.
- To assess the quality of the evidence found in systematic reviews, we follow the guidelines of the GRADE (Grading of Recommendations Assessment, Development and Evaluation).
- When applicable, publication bias will be assessed.

### Evaluating the reliability of results

This systematic review protocol follows the standards contained in the tool methodological PRISMA-P (Preferred Reporting Items for Systematic Review and Meta-Analysis Protocols) [18, 19]—check the PRISMA checklist in Additional file 2.

## Discussion

Data will be extracted using standardized forms with the following information: scope of study, sample characteristics, objectives, design, data collection, methods of analysis, data source, and results.

Considering the heterogeneity of the definitions of abuse, disrespect, and mistreatment of women in labor or abortion, it may not be possible to carry out the meta-analysis of the frequency of events reported in the included articles. Events reported by the original articles will be classified according to a typology of abuse, disrespect, and maltreatment in the labor or abortion process described by Bohren et al. [1].

## Additional file

Additional file 1: Table S1. Types of abuse and disrespect of women in the process of delivery and/or abortion. (PDF 190 kb)
Additional file 2: PRISMA-P (Preferred Reporting Items for Systematic review and Meta-Analysis Protocols) 2015 checklist: recommended items to address in a systematic review protocol. (DOC 83 kb)

## Footnotes

1 World Health Organization (WHO). The prevention and elimination of disrespect and abuse during facility-based childbirth, 2014. <http://apps.who.int/iris/bitstream/10665/134588/1/WHO_RHR_14.23_eng.pdf?ua=1>.
