# Supplementary material for: Abuse and disrespect in childbirth process and abortion situation in Latin America and the Caribbean—systematic review protocol
Source: Syst Rev. 2017 Aug 3;6:152. doi: 10.1186/s13643-017-0516-5 (PMC5541749; doi:10.1186/s13643-017-0516-5)
Supplement: Additional file 1: Table S1. — Types of abuse and disrespect of women in the process of delivery and/or abortion. (PDF 190 kb) [file 13643_2017_516_MOESM1_ESM.pdf]

Additional file 1: Table 1
Table 1: Types of abuse and disrespect of women in the process of delivery and/or abortion[1]

Third order Terms	Second order Terms	First Order Terms
Physical abuse	Use of force<br>Physical restraint	Women beaten, slapped, kicked or pinched during delivery<br>Women physically restrained to the bed or gagged during delivery
Sexual abuse	Sexual Abuse	Sexual abuse or rape
Verbal abuse	Harsh language<br>Threats and blaming	Harsh or rude language<br>Judgmental or accusatory comments<br>Threats of withholding treatment or por outcomes<br>Blaming for por outcomes
Stigma and discrimination	Discrimination based on sociodemographic characteristics<br>Discrimination based on medical conditions	Discrimination based on ethnicity/race/religión<br>Discrimination based on age<br>Discriminaton based on socioeconomic status<br>Discriminaton based on HIV status
Failure to meet professional standards of care	Lack of informed consent and confidentiality<br>Physical examinations and procedures<br>Neglect and abandonment	Lack of informed consent process<br>Breaches of confidentiality<br>Painful vaginal exams<br>Refusal to provide pain relief<br>Performance of unconsented surgical operations<br>Neglect, abandonment or long delays<br>Skilled attendant absent at time of delivery
Poor rapport between women and providers	Ineffective communication<br>Lack of supportive care<br>Loss of autonomy	Poor communication<br>Dismissal of women's concerns<br>Language and interpretation issues<br>Poor staff attitudes<br>Lack of supportive care from health workers<br>Denial or lack of birth companions<br>Women treated as passive participants during childbirth<br>Denial of food, fluids or mobility<br>Lack of respect for women's preferred birth positions<br>Denial of safe traditional practices<br>Objectification of women<br>Detainment in facilities

Source: Data adapted from Bohren et al (2015).

[1] The information in this table were taken from a similar table developed by Bohren et al (2015), from where we deliberately remove information about conditions and restrictions of the health system for not belonging to approach this review.
